# The single nucleotide polymorphism rs4986790 (c.896A>G) in the gene *TLR4* as a protective factor in corona virus disease 2019 (COVID-19)

**DOI:** 10.3389/fimmu.2024.1355193

**Published:** 2024-02-16

**Authors:** Christoph Zacher, Kristina Schönfelder, Hana Rohn, Winfried Siffert, Birte Möhlendick

**Affiliations:** ^1^ Institute of Pharmacogenetics, University Hospital Essen, University of Duisburg-Essen, Essen, Germany; ^2^ Department of Nephrology, University Hospital Essen, University of Duisburg-Essen, Essen, Germany; ^3^ Department of Infectious Diseases, University Hospital Essen, University of Duisburg-Essen, Essen, Germany

**Keywords:** SARS-CoV-2, TLR4, COVID-19, polymorphism, rs4986790, disease severity, prognostic marker, IL-6

## Abstract

**Background and aims:**

Several factors, such as hypertension and diabetes mellitus, are known to influence the course of coronavirus disease 2019 (COVID-19). However, there is currently little information on genetic markers that influence the severity of COVID-19. In this study, we specifically investigated the single nucleotide polymorphism (SNP) rs4986790 in the *TLR4* gene to identify a universal marker for preclinical prediction of COVID-19 disease progression.

**Methods:**

We analyzed the influence of demographics, pre-existing conditions, inflammatory parameters at the time of hospitalization, and *TLR4* rs4986790 genotype on the outcome of COVID-19 in a comprehensive cohort (N = 1570). We performed multivariable analysis to investigate the impact of each factor.

**Results:**

We confirmed that younger patient age and absence of pre-existing conditions were protective factors against disease progression. Furthermore, when comparing patients with mild SARS-CoV-2 infection with patients who required hospitalization or intensive care or even died due to COVID-19, the AG/GG genotype of *TLR4* rs4986790 was found to be a protective factor against COVID-19 disease progression (OR: 0.51, 95% CI: 0.34 - 0.77, *p* = 0.001). In addition, we demonstrated that low levels of interleukin-6 (IL-6) and procalcitonin (PCT) had a favorable effect on COVID-19 disease severity. In the subsequent multivariable analysis, we confirmed the absence of cardiovascular disease, low levels of IL-6 and PCT, and *TLR4* rs4986790 AG/GG genotypes as independent predictors of potential hospitalization and reduction of severe or fatal disease course.

**Conclusion:**

In this study, we identified an additional genetic factor that may serve as an invariant predictor of COVID-19 outcome. The *TLR4* rs4986790 AG/GG genotype reduced by half the risk of COVID-19 patients requiring hospitalization, intensive care or to have a fatal outcome. In addition, we were able to confirm the influence of previously known factors such as pre-existing conditions and inflammatory markers upon the onset of disease on the course of COVID-19. Based on these observations, we hereby provide another prognostic biomarker that could be used in routine diagnostics as a predictive factor for the severity of COVID-19 prior to SARS-CoV-2 infection.

## 1 Introduction

Toll-like receptors (TLRs) recognize specific structures called pathogen-associated molecular patterns (PAMPs) and viral proteins on the surface of pathogens such as bacteria and viruses. There exist ten different variants of TLRs, which are located on the cell membrane as well as in endosomes. TLRs are expressed on both adaptive and innate immune cells, including T cells, B cells, dendritic cells, macrophages, and natural killer cells. Each TLR recognize specific structures. TLR3 recognizes double-stranded RNA, TLR4 recognizes lipopolysaccharides, and TLR7/8 recognize single-stranded RNA (1, 2).

Two different signaling pathways are known for TLR4. The first is via myeloid differentiation primary response 88 (MyD88), while the second pathway is via TIR-domain-containing adapter-inducing interferon-β (TRIF). This results in the activation of nuclear factor κ-light-chain-enhancer of activated B cells (NF-κB) and interferon regulatory factors (IRFs), leading to the production of type-1 IFN and pro-inflammatory cytokines such as interleukin-1 (IL-1), interleukin-6 (IL-6), interleukin-12 (IL-12), and tumor necrosis factor-α (TNF-α) (3, 4). The production of pro-inflammatory cytokines and interferons is critical in fighting viral infections. The spike protein of SARS-CoV-2 acts as a ligand for TLR4, which induces a strong protein-protein interaction. This can lead to overactivation of TLR4, resulting in a prolonged or excessive immune response (5, 6).

Excessive levels of cytokines can lead to dysregulation of the immune response, resulting in cytokine storm and cytokine release syndrome. This systemic inflammatory syndrome is characterized by life-threatening levels of circulating cytokines and hyperactivation of immune cells. In the worst case, this exaggerated immune response can lead to multiple organ failure with a fatal outcome of COVID-19 (7). Severe SARS-CoV-2 infection is characterized primarily by elevated levels of IL-6, but also IL-8, IL-10, TNF-α and interferon-γ (IFN-γ). In particular, IL-6 has been associated with a significantly higher mortality and may serve as an indicator for disease prognosis and the severity of in COVID-19 (8).

Receptor activity and sensitivity may be influenced by genetic variation. The single nucleotide polymorphism (SNP) rs4986790 in the *TLR4* gene has been frequently described in the literature (9–12). The SNP is located outside the ligand binding domain of TLR4 and therefore does not affect LPS binding. However, it causes a local conformational change that affects folding, cell surface expression levels, protein stability, and interaction with downstream messenger proteins. This results in a twofold reduction of functional TLR4 (13). In addition to the well-known risk factors for COVID-19, including older age, immunosuppression, chronic pulmonary, hepatic, renal, neurological, cardiovascular disease, and diabetes mellitus, some patients without these conditions also showed severe and fatal outcomes (14). In previous studies, we have shown that genetic factors also influence disease progression. The *ACE2* rs2285666 GG genotype or G-allele were associated with an almost twofold increased risk of infection and a threefold increased risk of a severe or fatal outcome of COVID-19 (15). We also demonstrated that the *GNB3* c.825C>T (rs5443) TT genotype is protective against COVID-19 fatality (16).

Because TLR4 plays a pivotal role in binding to the SARS-CoV-2 spike protein and regulating downstream inflammatory responses (17), we investigated the influence of the SNP rs4986790 (c.896A>G) in the *TLR4* gene and the associated expression of interleukin-6 as an inflammatory mediator in the current study.

## 2 Methods

### 2.1 Study participants, recruitment and patient outcome

The study was approved by the Ethics Committee of the Medical Faculty of the University of Duisburg-Essen (20-9230-BO) and was conducted in collaboration with the West German Biobank (WBE; 20-WBE-088). Written informed consent was obtained from all patients enrolled in the study.

Enrollment started on March 11, 2020, and ended on May 18, 2021. A total of 1570 SARS-CoV-2 positive patients with at least one positive reverse transcription polymerase chain reaction (RT-PCR) test result were included. Follow-up was completed on June 30, 2021, at which time all patients had been discharged from the hospital or had died during the study. The study included 660 female patients (42.0%) and 910 male patients (58.0%). Age ranged from 18 to 99 years, with a median of 62.0 years. Patients were unvaccinated and did not receive targeted antibody therapy during the observation period.

The categorization was based on ECDC (European Centre for Disease Prevention and Control, 2021) criteria and considered the worst achieved condition after SARS-COV-2 infection. The ‘mild’ group included outpatients with no or mild symptoms not requiring hospitalization (N = 205, 13.1%). The ‘hospitalized’ group encompassed all patients who were hospitalized but did not require intensive care at any time (N = 760, 48.4%). To be included in the ‘severe’ group, patients had to be admitted to an intensive care unit due to SARS-COV-2 infection and/or intubated due to respiratory failure or indirect sequelae of SARS-COV-2 infection (N = 292, 18.6%). The ‘fatal’ group consisted of patients who died of SARS-COV-2 infection despite receiving medical care after admission (N = 313, 19.9%).

For a comprehensive analysis, we included additional data from the patients’ medical history. This included conditions such as hypertension, cardiovascular disease, diabetes mellitus, and laboratory values of interleukin-6 (IL-6) and procalcitonin (PCT).

### 2.2 Genotyping of *TLR4* rs4986790 (c.896A>G, p.Asp299Gly)

Genomic DNA was extracted from 200 μl EDTA-treated blood using the QIAamp^®^ DNA Blood Mini Kit (Qiagen, Hilden, Germany). Polymerase chain reaction (PCR) was performed using 2 μl genomic DNA and 30 μl Taq DNA-Polymerase 2x Master Mix Red (Ampliqon, Odense, Denmark) with the following conditions: initial denaturation at 95°C for 5 min; 36 cycles of denaturation at 95°C for 30 s, annealing at 60°C for 30 s, and extension at 72°C for 30 s each; final extension at 72°C for 10 min (forward primer: 5′ AGA GGG CCT GTG CAA TTT GA 3’ and reverse primer 3′ TCC CAC CTT TGT TGG AAG TGA 5′). PCR products were purified and *TLR4* rs4986790 genotypes were determined by Sanger sequencing.

### 2.3 Statistical analyses

Correlation of demographics (sex, medical history) and outcome of COVID-19 was calculated using Pearson’s chi-squared statistic (χ^2^) with Baptista-Pike method for odds ratio (OR) and 95% confidence interval (CI). One-way analysis of variance (ANOVA) was performed using the Kruskal-Wallis test with Dunn’s multiple comparison to assess the influence of age, laboratory parameters, and pre-existing conditions on COVID-19 severity. Receiver operating characteristic (ROC) analysis and Youden’s J statistic were used to calculate thresholds for laboratory values correlating with severe or fatal disease.

Hardy-Weinberg equilibrium (HWE) was calculated using Pearson’s chi-squared goodness-of-fit test (χ^2^), and samples were considered to deviate from HWE at a significance level of *p* < 0.05.

The number of patients with the *TLR4* rs4986790 GG genotype was small (N = 7). For this reason, we pooled the patients with *TLR4* rs4986790 AG and GG genotypes into one group.

For genetic association, we calculated OR and 95% CI by Pearson’s chi-squared statistic (χ^2^) using the Baptista-Pike method for OR and 95% CI, respectively. *P*-values are two-tailed and values < 0.05 were considered significant. Multivariable analysis was used to estimate the independence of age, sex, medical history, laboratory parameters, and *TLR4* rs4986790 genotype by stepwise Cox regression (likelihood ratio test, backwards).

## 3 Results

From March 11, 2020, to June 30, 2021, 1570 SARS-CoV-2 positive patients were enrolled and evaluated to determine the association between the rs4986790 SNP in the *TLR4* gene and the severity of COVID-19. We also analyzed IL-6 (N = 1060) and PCT (N = 1365) levels in all patients who had a laboratory determination of these parameters at the time of hospital admission. The demographic and clinical characteristics of the patients are summarized in **Table 1**.

**Table 1:** Demographics, clinical indications, and outcome of disease in SARS-CoV-2-positive patients.

Characteristics	All patients (N = 1570)	Mild (N = 205)	Hospitalized (N = 760)	Severe (N = 292)	Fatal (N = 313)	*p*-value
Age - years	62.0 (49.0 - 76.0)	47.0 (34.5 - 64.0)	61.0 (48.3 - 76.0)	59.0 (50.0 - 70.0)	71.0 (59.8 - 82.0)	< 0.0001
Male sex	910 (58.0)	107 (52.2)	416 (54.7)	185 (63.4)	202 (64.5)	0.002
Medical History						
Diseases of cardiovascular system^1^	547 (34.8)	11 (5.4)	257 (33.8)	111 (38.0)	168 (53.7)	< 0.0001
Arterial Hypertension	748 (47.6)	29 (14.1)	373 (49.1)	149 (49.1)	197 (62.9)	< 0.0001
Diabetes Mellitus	404 (25.7)	14 (6.8)	214 (28.2)	76 (26.0)	100 (31.9)	0.001
Inflammatory markers						
Interleukin-6 (high) pg/mL^2^	736 (69.4)	24 (25.8)	301 (62.3)	195 (80.9)	216 (88.5)	< 0.0001
Procalcitonin (high) ng/mL^3^	779 (57.1)	16 (15.7)	291 (42.9)	205 (73.3)	267 (87.8)	< 0.0001

Classification according to the ECDC COVID-19 surveillance report. Sex and medical history are expressed as absolute number and percentages. All other values are given as median and interquartile range (IQR). Only data that could be obtained from the patients' medical record were included in the calculation. Both pre-existing conditions and laboratory values for IL-6 and PCT were not fully documented; only values that were collected were included in the calculation. Laboratory values were taken at the time of hospital admission, which in the majority of cases corresponded to the early onset of COVID-19.

^1^e.g. myocardial infarction, coronary heart disease, but not arterial hypertension. ^2^Interleukin-6 (high) refers to patients in whom the calculated threshold of 18.75 pg/mL was exceeded. ^3^Procalcitonin (high) refers to patients in whom the calculated threshold of 0.075 ng/mL was exceeded. Units: pg/mL = picogram per milliliter, ng/mL = nanogram per milliliter.

With increasing disease severity the number of male (*p* = 0.002) and older (*p* < 0.0001) patients and the frequency of pre-existing conditions such as arterial hypertension (*p* < 0.0001), other cardiovascular diseases (*p* < 0.0001) and diabetes mellitus (*p* = 0.001) increased significantly.

**Figure 1:**
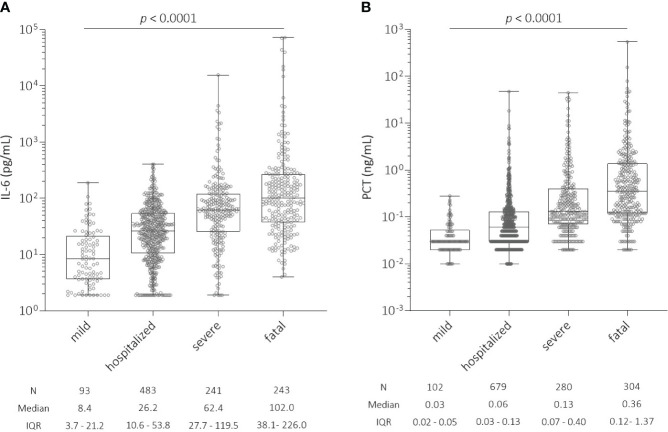
**(A)** Distribution of interleukin-6 (IL-6) levels (pg/mL) at the time of hospital admission among all patients with SARS-CoV-2 infection according to COVID-19 severity. There was a significant increase in IL-6 levels with COVID-19 severity (*p* < 0.0001): ‘mild’ (N = 93, median = 8.4, IQR = 3.7 - 21.2); ‘hospitalized’ (N = 483, median = 26.2, IQR = 10.6 - 53.8); ‘severe’ (N = 241, median = 62.4, IQR = 27.7 - 119.5); and ‘fatal’ (N = 243, median = 102.0, IQR = 38.1 - 226.0). **(B)** Distribution of procalcitonin (PCT) levels (ng/mL) at the time of hospital admission among all patients with SARS-CoV-2 infection according to COVID-19 severity. There was a significant increase in PCT levels with COVID-19 severity (*p* < 0.0001): ‘mild’ (N = 102, median = 0.03, IQR = 0.02 - 0.05); ‘hospitalized’ (N = 679, median = 0.06, IQR = 0.03 - 0.13); ‘severe’ (N = 280, median = 0.13, IQR = 0.07 - 0.40); and ‘fatal’ (N = 304, median = 0.36, IQR = 0.12 - 1.37). IL-6, interleukin-6; PCT, procalcitonin; IQR, interquartile range.

Using ROC analysis, we estimated a threshold for IL-6 and PCT levels above which the risk of hospitalization and severe or fatal outcome of COVID-19 increased. For IL-6, levels above 18.75 pg/mL blood appear to be critical for a patient to at least be hospitalized or to have a severe or fatal outcome. For PCT, we found that patients with levels above 0.075 ng/mL blood were more likely to be hospitalized or to have a severe or fatal outcome of COVID-19. Patients with a mild or asymptomatic course in the ‘mild’ group had the lowest IL-6 levels (median = 8.4, N = 93, IQR = 3.7 - 21.2), which significantly increased with disease severity (*p* < 0.0001, **Figure 1A**), while the highest median IL-6 levels were found in the ‘fatal’ group (median = 102.0, N = 243, IQR = 38.1 - 226.0). We also observed a significant increase in PCT levels with disease severity (*p* < 0.0001, **Figure 1B**). Median PCT levels were 10-fold higher in the ‘fatal’ group (median = 0.36, N = 304, IQR = 0.12 - 1.37) compared to the ‘mild’ group (median = 0.03, N = 102, IQR = 0.02 - 0.05).

### 3.1 *TLR4* rs4986790 as a protective factor against severe course of COVID-19

Overall, the observed genotypes for *TLR4* rs4986790 were consistent with HWE in patients with ‘mild’ (*p* = 0.20), ‘hospitalized’ (*p* = 0.16), ‘severe’ (*p* = 0.37), and ‘fatal’ (*p* = 0.17) SARS-CoV-2 infection. The genotype distribution for all patients according to the severity of SARS-CoV-2 infection is shown in **Table 2**. Notably, we observed very similar rs4986790 G-allele frequencies (4.0 - 6.0%) in all groups except in patients with ‘mild’ SARS-CoV-2 infection (8.0%). We assessed, whether carriers of the G-allele or the GG genotype might be better protected against the need for hospitalization or against severe or fatal disease outcome. We found a significant association for protection in rs4986790 AG or GG genotype carriers comparing all patients (‘hospitalized’, ‘severe’ and ‘fatal’) with COVID-19 with those with ‘mild’ or asymptomatic SARS-CoV-2 infection (OR: 0.51, 95% CI: 0.34 - 0.77; *p* = 0.001, **Table 3**).

**Table 2:** *TLR4* rs4986790 (c.896A>G) genotype distribution among all patients with SARS-CoV-2 infection according to the severity of COVID-19.

	All patients (N = 1570)	Mild (N = 205)	Hospitalized (N = 760)	Severe (N = 292)	Fatal (N = 313)
*TLR4* rs4986790 AA	1410 (89.8 %)	171 (83.4 %)	698 (91.8 %)	258 (88.4 %)	283 (90.4 %)
*TLR4* rs4986790 AG	153 (9.7 %)	34 (16.6 %)	59 (7.8 %)	32 (11.0 %)	28 (8.9 %)
*TLR4* rs4986790 GG	7 (0.4 %)	0 (0.0 %)	3 (0.4 %)	2 (0.7 %)	2 (0.6 %)
minor allele frequency (G)	0.05	0.08	0.04	0.06	0.05

**Table 3:** Protective factors against COVID-19 disease progression comparing patients with mild SARS-CoV-2 infection with patients who required hospitalization or intensive care treatment or had a fatal outcome due to COVID-19.

Factor	Univariate analysis		Multivariable analysis	
	OR [95 % CI]	*p*-value	OR [95 % CI]	*p*-value
Age (≤ 62 years)	0.34 [0.24 - 0.47]	< 0.0001	0.54 [0.30 – 1.00]	0.047
Sex (female)	0.76 [0.57 - 1.03]	0.07	NS	NS
Absence of				
Diseases of the cardiovascular system^1^	0.16 [0.09 - 0.31]	*<* 0.0001	0.23 [0.09 - 0.57]	0.002
Arterial Hypertension	0.30 [0.20 - 0.46]	*<* 0.0001	NS	NS
Diabetes mellitus	0.34 [0.20 - 0.61]	*<* 0.0001	NS	NS
Inflammatory markers				
Interleukin-6 (< 18.75 pg/mL)	0.13 [0.08 - 0.20]	*<* 0.0001	0.21 [0.12 - 0.36]	*<* 0.0001
Procalcitonin (< 0.0075 ng/mL)	0.12 [0.07 - 0.21]	*<* 0.0001	0.27 [0.14 - 0.52]	*<* 0.0001
***TLR4* rs4986790 AG/GG genotype**	0.51 [0.34 - 0.77]	0.001	0.47 [0.23 - 0.96]	0.039

^1^e.g. myocardial infarction, coronary heart disease, but not arterial hypotension. Units: pg/mL = picogram per milliliter, ng/mL = nanogram per milliliter. OR, odds ratio; CI, confidence interval; NS, not significant in stepwise multivariable analysis.

In univariate analyses, we also found that age (≤ 62 years, OR: 0.34, 95% CI: 0.24 - 0.47; *p* < 0.0001), absence of pre-existing conditions [cardiovascular disease (OR: 0.16, 95% CI: 0.09 - 0.31; *p* < 0.0001), arterial hypertension (OR: 0.30, 95% CI: 0.20 - 0.46; *p* < 0.0001), diabetes mellitus (OR: 0.34, 95% CI: 0.20 - 0.61; *p* < 0.0001)], low levels of the two selected inflammatory markers IL-6 (OR: 0.13, 95% CI: 0.08 - 0.20; *p* < 0.0001) and PCT (OR: 0.12, 95% CI: 0.07 - 0.21; p < 0.0001) were significant predictors of protection against hospitalization due to SARS-CoV-2 infection or severe or fatal course of COVID-19.

To estimate the independence of the *TLR4* rs4986790 AG or GG genotype as a protective factor compared to the other predictive parameters, namely, age, pre-existing conditions, IL-6 and PCT, we performed multivariable analysis by stepwise Cox regression. We compared the ‘mild’ group with all others (‘hospitalized’, ‘severe” and ‘fatal’) to estimate which factors are independent predictors of protection against SARS-CoV-2 infection requiring hospitalization or against COVID-19 severe or fatal outcome. We observed that absence of cardiovascular disease (OR: 0.23, 95% CI: 0.09 - 0.57; *p* = 0.002), low IL-6 (OR: 0.21, 95% CI: 0.12 - 0.36; *p* < 0.0001) and PCT levels (OR: 0.27, 95% CI: 0.14 - 0.52; *p* < 0.0001) and *TLR4* rs4986790 AG or GG genotype (OR: 0.47, 95% CI: 0.23 - 0.96; *p* = 0.039) remained independent protective factors (**Table 3**).

## 4 Discussion

Specifically, we observed that the AG or GG genotype of rs4986790 in the *TLR4* gene was associated with protection against hospitalization, intensive care or death from COVID-19. Previously, two other groups investigated the SNP *TLR4* rs4986790 and its association with COVID-19 severity.

In contrast to our observations, Taha *et al.* found that the G-allele of rs4986790 was associated with a significantly higher risk of severe COVID-19 (N = 300, OR = 3.14, 95% CI = 2.02 - 4.88, *p* < 0.001) in a cohort of Egyptian patients (18). The authors also observed a significant increase in IL-6 levels in carriers of the rs4986790 G-allele, which we could not confirm in our study (data not shown).

The completely opposite effect observed in our study, could be due to the different allele distribution in our European (MAF = 0.05) and the Egyptian cohort (MAF = 0.18) in the study by Taha *et al.* On the other hand, we examined a much larger cohort in our study, which could also have led to differences in the observations.

A second study investigated the possible contribution of SNPs in *TLR2* and *TLR4* genes to COVID-19 disease severity and prognosis in an European population (N = 249) consisting mainly of Greek patients with SARS-CoV-2 infection (19). These authors found no significant association of *TLR4* rs4986790 with COVID-19 disease severity, although the minor allele frequency was comparable to that observed in our study (0.04 vs. 0.05). They showed a significantly increased risk of severe COVID-19 in carriers of *TLR2* rs57443708 and/or *TLR4* rs4986791 variants. Although there are discrepancies between our study and the other studies, all analyses show that polymorphisms in the Toll-like receptor gene family appear to have an important influence on the course of COVID-19. The direct functional effects of the SNPs are still largely unclear and need to be evaluated in further studies.

We confirmed that cardiovascular disease, arterial hypertension, and diabetes mellitus are associated with an increased rate of severe progression and fatality in COVID-19 patients, as observed in several other studies. In univariate analysis, we also confirmed the influence of patient age as a prognostic factor (20, 21). Furthermore, we investigated the influence of interleukin-6 and procalcitonin on the course of the disease. Our study showed an association between high levels of both IL-6 and PCT and a severe course or even death in COVID-19 patients at the time of hospital admission. Several other studies have demonstrated that the inflammatory markers IL-6 and PCT are predictors of in-hospital mortality (22–25).

In this study, only patients who were not vaccinated against COVID-19 were analyzed. The extent to which the prognostic parameters identified in this or other studies play a role in vaccinated patients remains unclear. Given the current evolution of the infection process, with the appearance of more infectious yet potentially harmless variants and thus fewer severe cases, additional factors influencing the course of the disease may need to be identified. In this multivariable analysis, we were able to confirm a younger age, the absence of cardiovascular disease, the G-allele of *TRL4* rs4986790 and low levels of the inflammatory markers IL-6 and PCT upon hospital admission as independent protective factors against hospitalization, intensive care or fatal course of COVID-19. Time-independent factors that can be used to predict the course of COVID-19 are still very rare. For this reason, analysis of host genetics would be an important component that could be easily implemented in routine diagnostics.

## Data availability statement

The original contributions presented in the study are included in the article/supplementary materials. Further inquiries can be directed to the corresponding author.

## Ethics statement

The studies involving humans were approved by the Ethics Committee of the Medical Faculty of the University of Duisburg-Essen (20-9230-BO) and was conducted in collaboration with the West German Biobank (WBE; 20-WBE-088). Written informed consent was obtained from all patients enrolled in the study. The studies were conducted in accordance with the local legislation and institutional requirements. The participants provided their written informed consent to participate in this study.

## Author contributions

CZ: Data curation, Formal analysis, Investigation, Methodology, Resources, Visualization, Writing – original draft. KS: Resources, Writing – review & editing. HR: Data curation, Resources, Writing – review & editing. WS: Supervision, Validation, Writing – review & editing. BM: Conceptualization, Data curation, Formal analysis, Funding acquisition, Methodology, Project administration, Resources, Supervision, Validation, Writing – original draft, Writing – review & editing.
